# Distinctive Anatomical Patterns of the Mandibular Coronoid Process, Condyle, and Sigmoid Notch: Cone Beam Computed Tomography (CBCT) Imaging for Advanced Personal Identification

**DOI:** 10.7759/cureus.60978

**Published:** 2024-05-24

**Authors:** Jency Evanjelin P, Umamaheswari T.N

**Affiliations:** 1 Department of Oral Medicine and Radiology, Saveetha Dental College and Hospitals, Saveetha Institute of Medical and Technical Sciences, Saveetha University, Chennai, IND

**Keywords:** cbct imaging, sigmoid notch, condyle, mandibular coronoid process, anatomical patterns

## Abstract

Introduction: The human mandible plays a significant role in personal identification due to its unique morphological characteristics. The coronoid process, condyle, and sigmoid notch exhibit variations that can be utilized for forensic and anthropological purposes. This study aims to analyze the morphological diversities of these mandibular features in our ethnic group using cone beam computed tomography (CBCT) imaging.

Materials and methods: A retrospective analysis was conducted using 100 CBCT images obtained from the archives of the Department of Oral Medicine and Radiology. The images were captured using Carestream 9600 machines (Carestream Dental LLC, Atlanta, GA) with standard exposure parameters. Curved slicing screenshots were utilized for tracing the morphological variations of the coronoid process, condyle, and sigmoid notch.

Results: Out of 100 CBCT images analyzed (corresponding to 200 sides), the shape-wise distribution of the coronoid process revealed that a triangular shape was most commonly observed in 59% (118) of cases. The condyle exhibited a predominantly round shape in 38.7% (77) of cases based on shape parameter-wise distribution. Similarly, the sigmoid notch displayed a round shape in 40.5% (81) of cases based on shape-wise distribution.

Conclusion: Personal identification using radiographs has gained significance in the current scenario due to its feasibility. Cone beam computed tomography imaging has become a reliable and accurate method for revealing hidden details in forensic odontology, especially when antemortem records are available. This research sheds light on the morphological variations of the mandibular coronoid process, condyle, and sigmoid notch within our ethnic cohort, enhancing personal identification practices. Further research encompassing larger sample sizes and diverse populations would enhance the applicability of these findings in forensic and anthropological contexts.

## Introduction

The temporomandibular joint (TMJ) stands as one of the human body's most intricate and vital joints. It engages in critical functions such as mastication, speech, and swallowing through the coordinated action of both hard and soft tissues, including the mandibular condyle and the articular fossa of the temporal bone [1, 2]. The health and functionality of the TMJ are paramount for these vital activities, and any impairment can significantly impact quality of life. Temporomandibular joint dysfunction (TMD) is a multifactorial disorder characterized by a spectrum of symptoms, including pain in the joint, clicking or popping sounds, headaches, limited jaw movement, and muscle soreness [3]. The etiology of TMD is diverse, encompassing degenerative joint diseases, trauma, and functional overloads, which can lead to morphological changes in the joint components such as the articular disc, eminence, fossa, and mandibular condyle [4,5]. The TMJ is unique not only in its complexity but also in its growth and developmental aspects. The mandibular condyle, in particular, plays a crucial role in expressing mandibular growth and undergoes morphological changes influenced by age, gender, facial type, occlusal forces, functional load, malocclusion types, and differences between the right and left sides [6]. These factors can lead to variations in TMJ morphology, which are critical in understanding the development and progression of TMD [7]. As the population ages, degenerative changes in the TMJ become more prevalent, contributing to the morphologic alterations observed in elderly individuals. These changes can significantly affect the quality of life by impairing the basic functions of the TMJ [8]. Therefore, early detection and accurate assessment of TMJ morphology are crucial for diagnosing and managing TMD effectively. Panoramic radiography has been widely adopted as a simple, cost-effective method for evaluating the bony components of the TMJ. By employing the tomographic principle, it focuses on a specific plane of interest, rendering the TMJ and teeth in sharp detail while blurring adjacent structures [9].

Despite its utility, panoramic radiography has limitations in providing detailed, three-dimensional (3D) information about TMJ morphology [10]. Cone beam computed tomography (CBCT) has emerged as a superior imaging modality for assessing TMJ disorders, offering detailed 3D visualization of the joint's bony structures without the superimposition of surrounding tissues [11, 12]. Cone beam computed tomography imaging enables an in-depth examination of the morphological differences in the coronoid process, condyle, and sigmoid notch. These differences are crucial for grasping the pathophysiology of TMD and for identifying individuals within particular ethnic groups [13, 14]. The aim of this study is to explore the morphological variations of the TMJ components in our ethnic group using CBCT imaging. By doing so, it seeks to contribute to the broader understanding of TMD and its management, as well as to the forensic applications of TMJ imaging for personal identification [15].

## Materials and methods

Study design and population

This research was conducted as a descriptive, cross-sectional study at the Department of Oral Medicine and Radiology, Saveetha Dental College, Chennai, between 2019 and 2021. The study aimed to evaluate the morphological characteristics of the mandibular condyle, coronoid process, and sigmoid notch among patients attending the radiology clinic. Individuals were screened for eligibility based on their medical history and the reason for their CBCT imaging request.

Inclusion criteria

The study included subjects who underwent CBCT imaging as part of their diagnostic assessment for various oral health issues unrelated to TMD. Eligibility was contingent upon the CBCT scans being of high quality, without any distortions, and captured using correct and appropriate techniques. This criterion ensured the reliability of the morphological assessments to be conducted.

Exclusion criteria

Exclusion criteria were carefully defined to omit any CBCT scans that might compromise the study's integrity. Scans indicating developmental abnormalities of the mandible, signs of trauma, or any conditions impacting bone integrity were excluded. These conditions could potentially alter the morphology of the mandibular structures of interest and confound the study results.

Imaging methodology

All CBCT images were acquired using a Carestream 9600 machine (Carestream Dental LLC, Atlanta, GA), adhering to standardized exposure parameters with exposure of 120KV, 5mA, and 24.01s and with a voxel size of 300μmX300μmX300μm to ensure consistency across all scans. This uniformity is crucial for comparative analyses and ensures that variations in imaging conditions do not affect the study's outcomes. The Carestream 9600 is known for its high-resolution imaging capabilities, making it an ideal choice for detailed morphological studies of bone structures.

Analytical approach 

**Figure 1:**
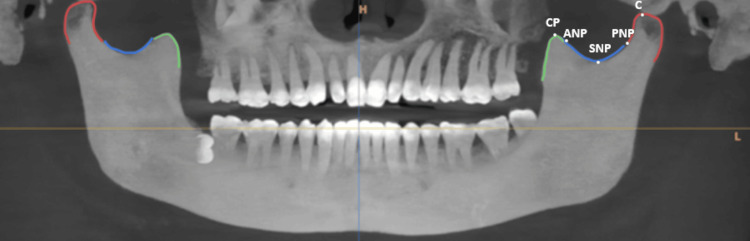
Tracing of the coronoid process, condyle, and sigmoid notch in a CBCT image CBCT: cone beam computed tomography; CP: coronoid process; ANP: anterior notch point; SNP: superior notch point; PNP: posterior notch point; C: condyle

For the analysis, each CBCT scan underwent a meticulous tracing process over the required anatomical landmarks, specifically focusing on the mandibular coronoid process, condyle, and sigmoid notch (Figure 1). The tracing process is as follows: coronoid process (CP): the anterior bony prominence; anterior notch point (ANP): the most anterior point of the sigmoid notch; superior notch point (SNP): the deepest point of the sigmoid notch; posterior notch point (PNP): the most posterior point of the sigmoid notch; condyle (C): the posterior bony prominence

This tracing was essential for accurately assessing the morphology of these structures. The tracings were then analyzed to identify morphological variations and patterns within the study population. This methodological approach allowed for a detailed examination of the structures of interest, providing insights into their morphological diversity within the sample population.

## Results

**Figure 2:**
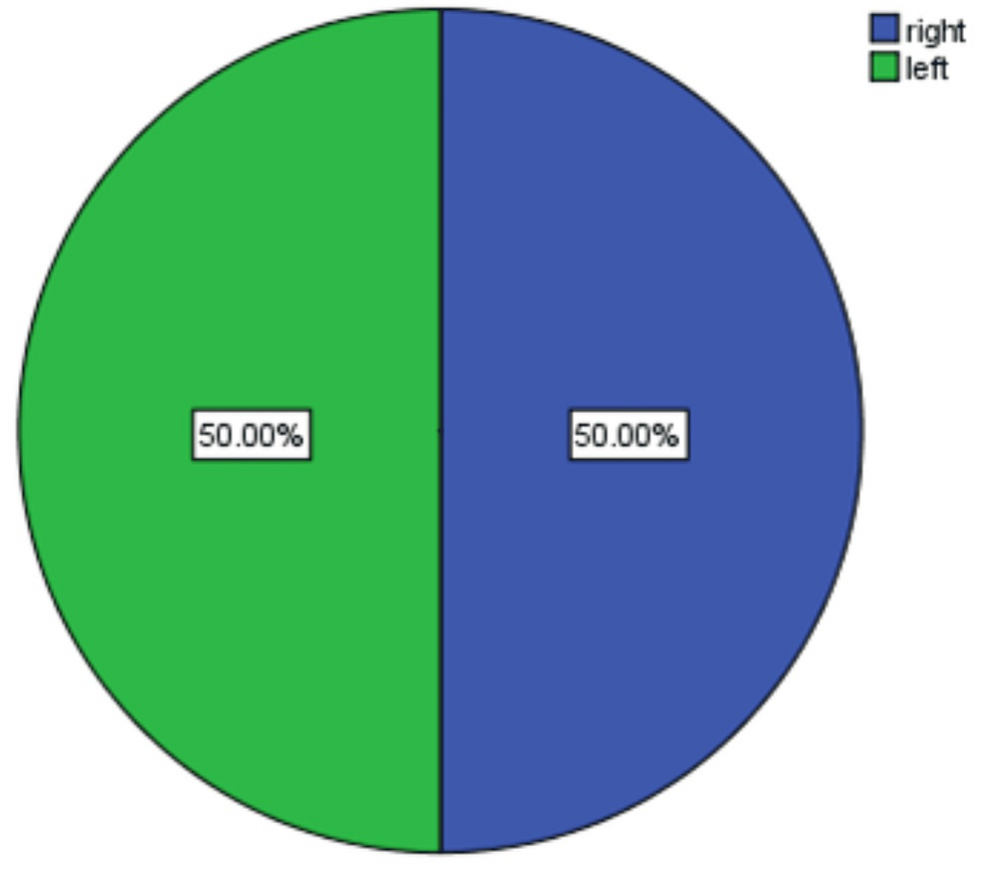
Equal distribution of the right and left sides of the TMJ TMJ: temporomandibular joint

In a comprehensive study involving 100 CBCT images, which effectively provided 200 distinct anatomical sides (right and left, with each category comprising 100 sides), a detailed examination was carried out to analyze the morphological variations of the coronoid process, condyle, and sigmoid notch on each side (Figure 2). This analysis was meticulously documented through various graphical representations, including graphs and figures, to illustrate the distribution and prevalence of different anatomical shapes across these bony structures.

**Figure 3:**
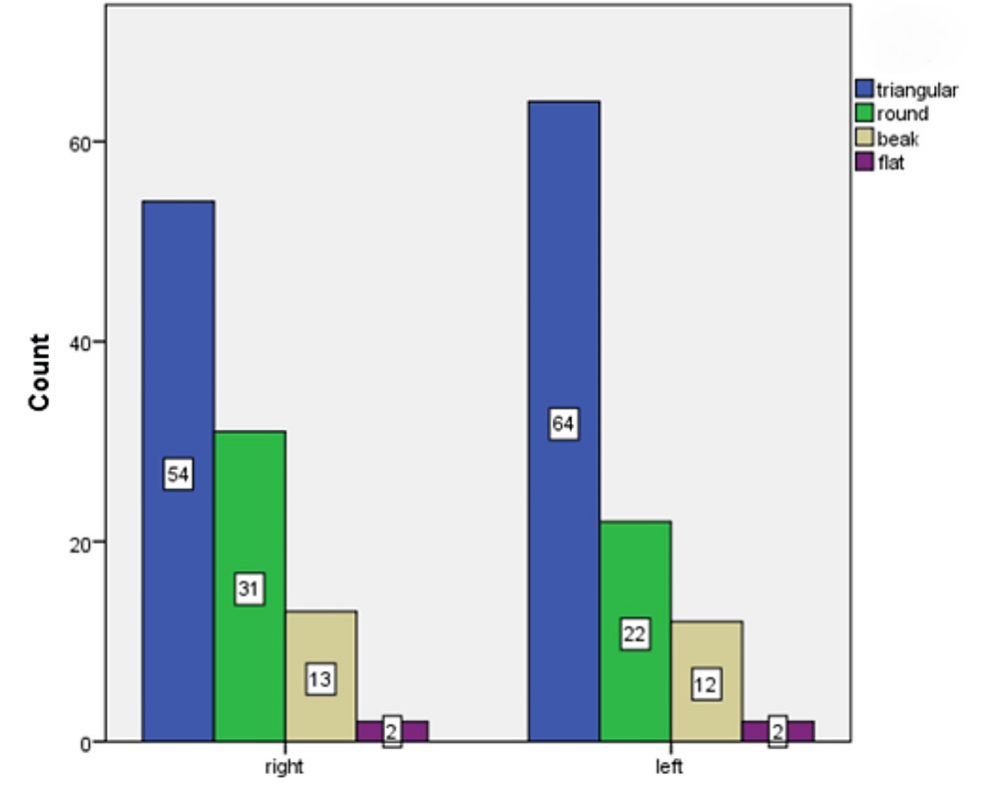
Shape and side-wise distribution of coronoid processes

**Figure 4:**
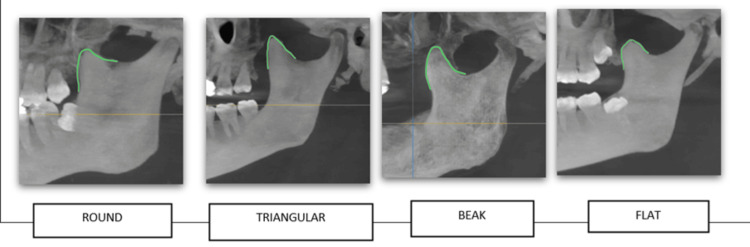
Tracing of morphological variation of the coronoid process in CBCT images CBCT: cone beam computed tomography

The investigation into the coronoid process, as depicted in Figure 3, highlighted a significant finding where the triangular shape emerged as the most common morphology, observed in 59% of cases (118 sides). Further delving into the side-wise distribution (Figure 4), it was noted that the left side predominantly featured distinction, which underscores the lateral variability in the morphology of the coronoid process.

**Figure 5:**
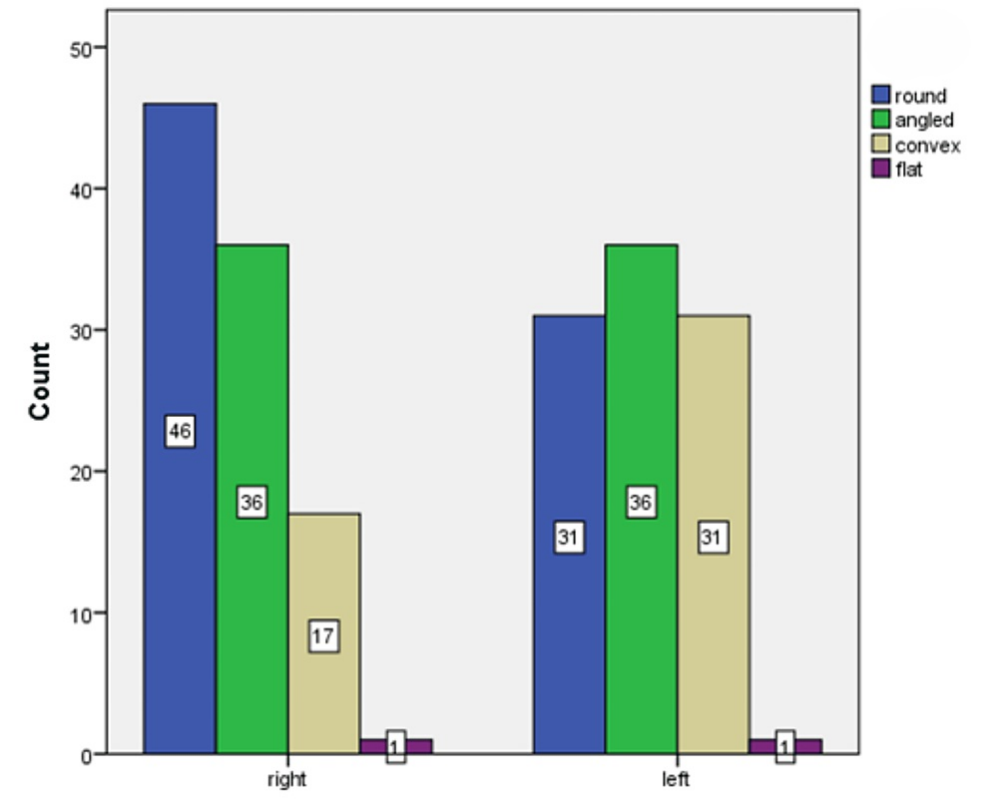
Shape and side-wise distribution of the condylar process

**Figure 6:**
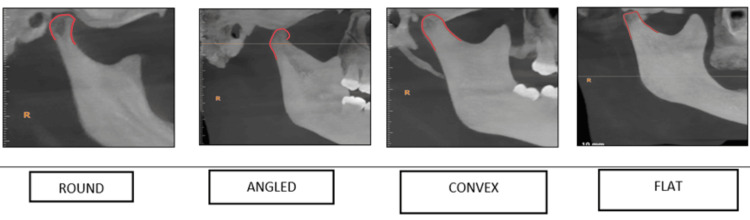
Tracing of the morphological variations of the condyle in CBCT images CBCT: cone beam computed tomography

When focusing on the condyle's shape distribution, Figure 5 revealed that the round shape was the most frequently observed, accounting for 38.7% (77 sides) of the sample. The side-wise distribution of the condyle's shape (Figure 6) presented a nuanced view where the round shape was predominantly seen on the right side and the angled shape was more commonly found on the left side. Additionally, convex and flat shapes were observed to be broadly distributed across both sides, indicating a diverse range of morphological presentations within the condyle.

**Figure 7:**
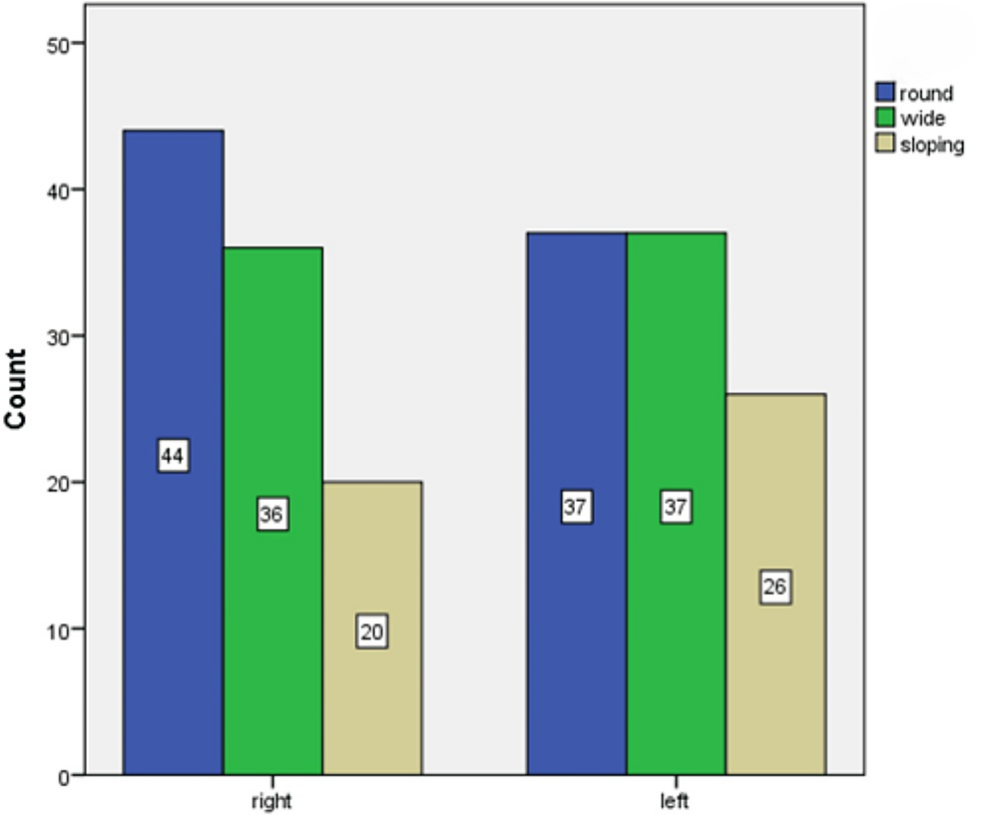
Shape and side-wise distribution of the sigmoid notch in CBCT images CBCT: cone beam computed tomography

**Figure 8:**
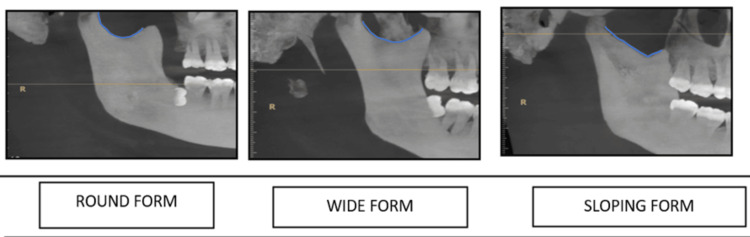
Tracing of morphological variations of the sigmoid notch in the mandible

The analysis extended to the sigmoid notch, with Figure 7 showcasing that the round shape was the most prevalent, observed in 40.5% (81 sides) of cases. The side-wise distribution of the sigmoid notch's shape (Figure 8) further elaborated on the lateral differences, where round and sloping shapes were more frequently seen on the right side and wide shapes were a common finding on the left side.

These findings, supported by the cited graphs and figures, provide a deep insight into the variability and prevalence of different anatomical shapes of the coronoid process, condyle, and sigmoid notch. The detailed examination not only highlights the diversity in morphological features across the jaw but also emphasizes the importance of considering these variations in clinical assessments, diagnosis, and treatment planning. The graphical representations serve as a visual aid to better understand the distribution patterns and prevalence rates of the various shapes, thereby enriching the study's results section with a clear and comprehensive overview of the morphological diversity observed in the sample.

## Discussion

The findings of this study underscore the significant morphological diversity present within the mandibular structures of the coronoid process, condyle, and sigmoid notch, as observed through CBCT imaging. This diversity not only has implications for clinical practice, particularly in the fields of orthodontics, oral surgery, and TMD but also holds considerable potential for forensic and anthropological identification. The discussion that follows integrates these findings with existing literature, highlighting their relevance and proposing directions for future research. The prevalence of a triangular shape in the coronoid process, as observed in this study, aligns with research indicating morphological variations in this structure can reflect genetic and environmental influences [16]. The side-wise distribution patterns noted, with triangular and flat shapes more common on the left and round and beak shapes on the right, suggest a lateral asymmetry that may be pertinent to understanding developmental anomalies and their clinical implications [17].

Regarding the condyle, the predominance of the round shape observed in our study mirrors findings from the study by Thompson and Popovich (2018), who noted that round and oval shapes were most common in a healthy population [18]. The variation in shape distribution between sides, particularly the prevalence of angled shapes on the left, could be indicative of adaptive responses to functional demands [19]. This adaptability underscores the condyle's role in accommodating different occlusal and functional scenarios, which is crucial for the management of TMD and orthodontic treatment planning [20]. The sigmoid notch's morphological diversity, particularly the commonality of round shapes, adds another layer to our understanding of TMJ anatomy. The side-wise distribution, with round and sloping shapes more prevalent on the right and wide shapes on the left, could reflect biomechanical adaptations or developmental asymmetries. These findings are in line with the work of Patel and Sandler (2021), who emphasized the importance of recognizing these variations for surgical planning and the treatment of TMJ disorders [21].

Furthermore, the application of CBCT imaging to uncover these morphological nuances has been validated by our findings, echoing the sentiments of Anderson et al. (2022) regarding CBCT's superiority in detailed anatomical assessments [22]. This imaging modality's ability to provide clear, three-dimensional views of bony structures makes it an invaluable tool in both clinical and forensic settings [23]. The potential forensic application of our findings, particularly in the context of personal identification, is significant. As suggested by Rodriguez and Wright (2024), the unique morphological characteristics of mandibular structures can serve as reliable markers for identifying individuals, especially when antemortem records are available [24]. This aspect of our research contributes to the growing body of literature advocating for the use of dental and skeletal markers in forensic anthropology [25]. The study's limitations include the retrospective nature of the analysis, which may affect the standardization of data, the focus on a specific ethnic group, limiting its applicability to other populations, and the focus on specific mandibular features, potentially overlooking other relevant anatomical structures.

## Conclusions

In conclusion, our study not only reinforces the value of CBCT imaging in assessing mandibular morphology but also highlights the clinical and forensic implications of understanding these morphological variations. Future studies should focus on building upon these results by including broader and more varied demographic groups to improve the applicability of the outcomes. Moreover, conducting studies over time could offer valuable information on the progression of these morphological features and their reactions to different clinical treatments.

## References

[1] Roy WA ( 2006). Temporomandibular disorders: an evidence-based approach to diagnosis and treatment. Phys Ther.

[2] Okeson JP (1993). Management of temporomandibular disorders and occlusion. https://intapi.sciendo.com/pdf/10.2478/aoj-1994-0029.

[3] Rongo R, Ekberg E, Nilsson IM (2021). Diagnostic criteria for temporomandibular disorders (DC/TMD) for children and adolescents: an international delphi study-part 1-development of axis I. J Oral Rehabil.

[4] Klasser GD, Greene CS (2009). The changing field of temporomandibular disorders: what dentists need to know. J Can Dent Assoc.

[5] Laskin DM (1969). Etiology of the pain-dysfunction syndrome. J Am Dent Assoc.

[6] Pullinger A G, Seligman D A. (2000). Quantification and validation of predictive values of occlusal variables in temporomandibular disorders using a multifactorial analysis. J Prosthet Dent.

[7] Koolstra JH (2002). Dynamics of the human masticatory system. Crit Rev Oral Biol Med.

[8] Tanaka E, Detamore MS, Mercuri LG (2008). Degenerative disorders of the temporomandibular joint: etiology, diagnosis, and treatment. J Dent Res.

[9] White SC, Pharoah MJ (2014). Oral Radiology: Principles and Interpretation.

[10] Alexiou K, Stamatakis H, Tsiklakis K (2009). Evaluation of the severity of temporomandibular joint osteoarthritic changes related to age using cone beam computed tomography. Dentomaxillofac Radiol.

[11] Honey OB, Scarfe WC, Hilgers MJ, Klueber K, Silveira AM, Haskell BS, Farman AG (2007). Accuracy of cone-beam computed tomography imaging of the temporomandibular joint: comparisons with panoramic radiology and linear tomography. Am J Orthod Dentofacial Orthop.

[12] Ahmed J, Sujir N, Shenoy N, Binnal A, Ongole R (2021). Morphological assessment of TMJ spaces, mandibular condyle, and glenoid fossa using cone beam computed tomography (CBCT): a retrospective analysis. Indian J Radiol Imaging.

[13] Hunter A, Kalathingal S (2013). Diagnostic imaging for temporomandibular disorders and orofacial pain. Dent Clin North Am.

[14] Bukhari SAA, Kambalyal PB, Reddy KA, Alhammadi MS, Shah SH (2019). Assessment of condylar volume and surface area in class I, class II and class III young adult Arab population. Dent Cadmos.

[15] Mustafi S, Sinha R, Roy D, Sen S, Maity S, Ghosh P (2019). Cone-beam computed tomography a reliable tool for morphometric analysis of the foramen magnum and a boon for forensic odontologists. J Forensic Dent Sci.

[16] Hersberger-Zurfluh MA, Motro M, Kantarci A, Will LA, Eliades T, Papageorgiou SN (2024). Genetic and environmental impact on mandibular growth in mono- and dizygotic twins during adolescence: a retrospective cohort study. Int Orthod.

[17] Liu YP, Behrents RG, Buschang PH (2010). Mandibular growth, remodeling, and maturation during infancy and early childhood. Angle Orthod.

[18] Solberg WK, Hansson TL, Nordström B (1985). The temporomandibular joint in young adults at autopsy: a morphologic classification and evaluation. J Oral Rehabil.

[19] Betti BF, Everts V, Ket JC, Tabeian H, Bakker AD, Langenbach GE, Lobbezoo F (2018). Effect of mechanical loading on the metabolic activity of cells in the temporomandibular joint: a systematic review. Clin Oral Investig.

[20] Choudhary A, Ahuja US, Rathore A, Puri N, Dhillon M, Budakoti A (2020). Association of temporomandibular joint morphology in patients with and without temporomandibular joint dysfunction: a cone-beam computed tomography based study. Dent Res J (Isfahan).

[21] Collins ED, Vossoughi F (2011). A three-dimensional analysis of the sigmoid notch. Orthop Rev (Pavia).

[22] de Boer EW, Dijkstra PU, Stegenga B, de Bont LG, Spijkervet FK (2014). Value of cone-beam computed tomography in the process of diagnosis and management of disorders of the temporomandibular joint. Br J Oral Maxillofac Surg.

[23] Issrani R, Prabhu N, Sghaireen MG (2022). Cone-beam computed tomography: a new tool on the horizon for forensic dentistry. Int J Environ Res Public Health.

[24] Cunha E, Ubelaker DH (2020). Evaluation of ancestry from human skeletal remains: a concise review. Forensic Sci Res.

[25] Hassanaly M, Caldas IM, Teixeir A, Pérez-Mongiovi D (2023). Application of CBCT technology in forensic odontology. Curr Forensic Sci.

